# Stress-Induced Anxiety- and Depressive-Like Phenotype Associated with Transient Reduction in Neurogenesis in Adult Nestin-CreER^T2^/Diphtheria Toxin Fragment A Transgenic Mice

**DOI:** 10.1371/journal.pone.0147256

**Published:** 2016-01-21

**Authors:** Sanghee Yun, Michael H. Donovan, Michele N. Ross, Devon R. Richardson, Robin Reister, Laure A. Farnbauch, Stephanie J. Fischer, Dieter Riethmacher, Howard K. Gershenfeld, Diane C. Lagace, Amelia J. Eisch

**Affiliations:** 1 Department of Psychiatry, The University of Texas Southwestern Medical Center, Dallas, TX, United States of America; 2 Department of Biomedical Sciences, Nazarbayev University School of Medicine, Astana, Kazakhstan; 3 Human Development and Health, School of Medicine, Southampton General Hospital, Southampton University, Southampton, United Kingdom; University of Amsterdam, NETHERLANDS

## Abstract

Depression and anxiety involve hippocampal dysfunction, but the specific relationship between these mood disorders and adult hippocampal dentate gyrus neurogenesis remains unclear. In both humans with MDD and rodent models of depression, administration of antidepressants increases DG progenitor and granule cell number, yet rodents with induced ablation of DG neurogenesis typically do not demonstrate depressive- or anxiety-like behaviors. The conflicting data may be explained by the varied duration and degree to which adult neurogenesis is reduced in different rodent neurogenesis ablation models. In order to test this hypothesis we examined how a transient–rather than permanent–inducible reduction in neurogenesis would alter depressive- and anxiety-like behaviors. Transgenic Nestin-CreER^T2^/floxed diphtheria toxin fragment A (DTA) mice (Cre+DTA+) and littermates (Cre+DTA-; control) were given tamoxifen (TAM) to induce recombination and decrease nestin-expressing stem cells and their progeny. The decreased neurogenesis was transient: 12 days post-TAM Cre+DTA+ mice had fewer DG proliferating Ki67+ cells and fewer DCX+ neuroblasts/immature neurons relative to control, but 30 days post-TAM Cre+DTA+ mice had the same DCX+ cell number as control. This ability of DG neurogenesis to recover after partial ablation also correlated with changes in behavior. Relative to control, Cre+DTA+ mice tested between 12–30 days post-TAM displayed indices of a stress-induced anxiety phenotype–longer latency to consume highly palatable food in the unfamiliar cage in the novelty-induced hypophagia test, and a depression phenotype–longer time of immobility in the tail suspension test, but Cre+DTA+ mice tested after 30 days post-TAM did not. These findings suggest a functional association between adult neurogenesis and stress induced anxiety- and depressive-like behaviors, where induced reduction in DCX+ cells at the time of behavioral testing is coupled with stress-induced anxiety and a depressive phenotype, and recovery of DCX+ cell number corresponds to normalization of these behaviors.

## Introduction

Depression and anxiety are devastating, prevalent psychiatric disorders diagnosed in a great number of people during their lifetime [1]. These disorders may have a shared etiology, since they have a high frequency of comorbidity and the symptoms can be improved by similar treatments [2, 3]. Another similarity between depression and anxiety is that they are often marked by impaired cognition [3–7], underscoring the involvement of limbic circuitry including the hippocampal formation. Structural evidence of hippocampal pathology has been widely reported. For example, humans diagnosed with major depressive disorder (MDD) have smaller hippocampi as visualized by *in vivo* imaging [8]. Also, unmedicated MDD subjects have a smaller hippocampus and fewer mature granule cells (GCs) in hippocampal dentate gyrus subregions as visualized by human *post-mortem* tissue analysis [9]. A potential explanation for these DG changes is that depression and anxiety may interfere with the process of DG neurogenesis, where local progenitors and neuroblasts give rise to new DG GC neurons throughout life [10–13], or that treatment for these disorders may stabilize or even increase DG neurogenesis [14–17]. Support for this “neurogenesis hypothesis” of affective and anxiety disorders comes from many studies, including human *post-mortem* studies showing that treatment with certain antidepressants increases the number of GCs and progenitors relative to non-treated MDD subjects [9, 18]. While it has long been postulated that adult DG neurogenesis contributes to the behavioral improvement seen after antidepressant administration, and reduced adult DG neurogenesis contributes to depressive- and anxiety-like behavior, as detailed below data from preclinical studies are conflicting, and more work is needed to test the proposed causal relationship.

Preclinically, adult DG neurogenesis appears to be required for antidepressant efficacy [15, 16, 19–24], and progenitors are a key target of antidepressant drugs [25–28]. However, preclinical studies do not agree on whether the disruption of neurogenesis (e.g. via ablation of progenitors, neuroblasts/immature neurons, and/or their progeny) leads to depressive- and anxiety-like behavior [21, 28–32]. Certainly some studies show that ablation of neurogenesis (via focal cranial irradiation, cytostatic agent methylazoxymethanol, or inducible hGFAP-thymidine kinase mice) results in depressive- and anxiety-like behavior: increased duration of immobility in the forced swim test, increased latency to feed in the novelty suppressed feeding test, and increased social avoidance [26, 33, 34]. However, other studies show that ablation of neurogenesis (via the same or distinct inducible ablation techniques, e.g. irradiation, Nestin-inducible Bax mice, Nestin-tk mice) does not result in depressive- and anxiety-like behavior [16, 17, 30, 35–37]. Results that appear to conflict can even appear within the same publication. For example, taking an opposite approach, a recent gain-of-function study showed that enhanced hippocampal neurogenesis (via transgenic, induced-deletion of Bax in nestin-expressing cells) did not change baseline depressive- or anxiety-like behaviors, but blunted depressive- and anxiety-like behaviors in a mouse model of stress [38]. Reasons for these discrepant results have been widely discussed [21, 28, 29], and include the varied duration and degree to which adult neurogenesis is disrupted, but no clear message has yet emerged from the field.

In regards to duration, it is notable that transient (vs. permanent) disruptions in neurogenesis correlate with or even cause temporary reduction in hippocampal-dependent learning and memory [36, 39–43]. Until recently, very few studies had examined whether transient induced disruptions in neurogenesis also correlate with or cause temporary reduction in mood and/or elevation in stress-induced anxiety. One recent study found that inducible, transgenic, transient reduction of surviving adult-generated neurons did not drive depression-related behaviors and stress-induced anxiety [30]. These data added weight to the argument that transiently decreased neurogenesis does not lead to a depressive-phenotype and stress-induced anxiety. However, surviving adult-generated neurons are only one functional component of the process of neurogenesis. Cells in “earlier” stages of neurogenesis, like progenitors and neuroblasts/immature neurons, serve both neurogenic and nonneurogenic functions in the adult DG [33, 42]. Given that these younger cells are key targets of antidepressant drugs [25, 27], more preclinical studies are needed to explore a whether a transient–rather than permanent–reduction in progenitors and neuroblasts/immature neurons–rather than surviving neurons–causes depressive-like behavior and stress-induced anxiety, and whether recovery of these cell numbers is associated with normalization of behavior.

Here we hypothesized that an induced, transient decrease in neurogenesis would be a relevant and effective way to assess a functional association between neurogenesis and depressive-like and stress-induced anxiety behavior. Therefore, we generated bigenic mice carrying the floxed *A chain diphtheria toxin fragment* (DTA) gene cassette [44] and the Nestin-CreER^T2^ inducible driver line [45]. After administration of tamoxifen (TAM), we examined cellular phenotypes and performance on a battery of behavioral tests over time. Our data show reduction in cells in early stages of neurogenesis (Ki67 immunoreactive[+] progenitors and doublecortin [DCX]+ neuroblasts and immature neurons) results in behaviors that reflect both stress-induced anxiety and depressive-like behavior: increased latency to consume highly palatable food in the unfamiliar cage in NIH test, and increased immobility in TST, respectively. However, at a later time point when DCX+ cell number normalized, stress-induced anxiety and depressive-like behavior were no longer evident. These findings highlight a functional association among adult neurogenesis, stress-induced anxiety, and depressive-like behaviors. We suggest induced reduction in DCX+ cells at the time of behavioral testing is associated with stress-induced anxiety and depressive-like behavior, while recovery of DCX+ cell number corresponds to normalization of these behaviors.

## Materials and Methods

### Animals and Ethics statement

Experiments were approved by the Institutional Animal Use and Care Committee at University of Texas Southwestern Medical Center (UTSW; APN 0960-07-02-1). Mice were group-housed in a UTSW vivarium accredited by the Association for Assessment and Accreditation of Laboratory Animal Care (AAALAC), and were kept on a 12 hr light/dark cycle with ad libitum access to food and water. Nestin-CreER^T2^, DTA and R26R-YFP mice have been published and characterized [44–46]. Briefly, Nestin-CreER^T2^ mice were generated by the Eisch Lab at UTSW [also available at Jackson Laboratory, strain C57BL/6-Tg(Nes-cre/ERT2)KEisc/J, stock number 016261], R26R-YFP were purchased from Jackson Laboratory [strain B6.129X1-Gt(ROSA)26Sortm1(EYFP)Cos/J, stock number 006148], and DTA mice were generated by the Riethmacher Laboratory [44]. Breeding for this study was performed at UTSW. Nestin-CreER^T2^ mice were bred with R26R-YFP mice, and the resulting mice (hemizygous both transgenes) were then crossed with DTA hemizygous mice, resulting in triple hemizygous mice for the three transgenes. While this breeding strategy allowed comparison of Nestin-CreER^T2^/DTA mice (Cre+DTA+) vs. littermate controls (Cre+DTA- or Cre-DTA-; control), it also resulted in only 25% of the litter being Cre+DTA+, with the remaining 75% of the litter used for control. Mice were genotyped by PCR using genomic DNA and primers previously published for Nestin-CreER^T2^, R26R-YFP, and DTA mice [44, 45].

### TAM administration

**Fig 1:**
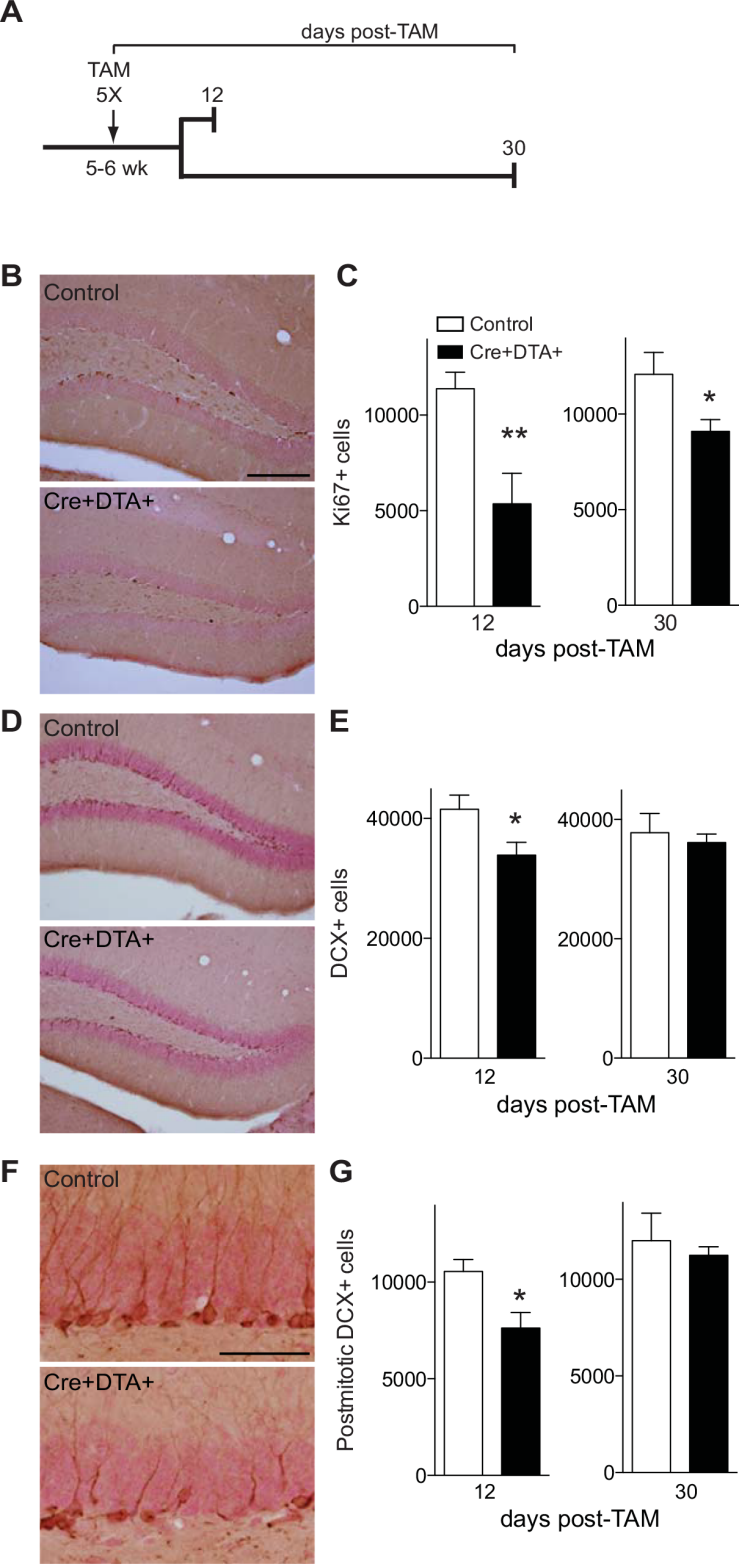
Inducible expression of DTA in nestin-lineage stem/progenitor cells decreases the number of Ki67+ and DCX+ cells 12 days (d) post-tamoxifen (TAM), but DCX+ cell number is normalized 30d post-TAM. **(A)** Experimental design of immunohistochemical study. TAM was administered to 5–6 week-old control or Cre+DTA+ mice for 5 consecutive days, and brains were collected 12d and 30d post-TAM. **(B)** Representative photomicrographs of the dentate gyrus from control and Cre+DTA+ mice 12d post-TAM stained with an antibody against Ki67. Scale bar = 200 um **(B,** applies to **B, D). (C)** Stereological quantification of Ki67+ cell number in the DG granule cell layer (GCL) 12d (control N = 5, Cre+DTA+ N = 4) and 30d (control N = 6, Cre+DTA+ N = 9) post-TAM. **(D)** Representative photomicrographs of the DG from control and Cre+DTA+ mice 12d post-TAM stained with antibody against DCX. **(E)** Stereological quantification of DCX+ cells in the DG GCL 12d (control N = 4, Cre+DTA+ N = 5) and 30d (control N = 6, Cre+DTA+ N = 7) post-TAM. **(F)** High magnification images of the DG from control and Cre+DTA+ mice 12d post-TAM stained with an antibody against DCX+. Scale bar = 50um. **(G)** Stereological quantification of postmitotic DCX+ cells in the DG GCL 12d (control N = 4, Cre+DTA+ N = 5) and 30d (control N = 6, Cre+DTA+ N = 7). Data are mean±SEM,.**p<0.01, *p<0.05 by unpaired, two-tailed Student’s t-test.

**Fig 2:**
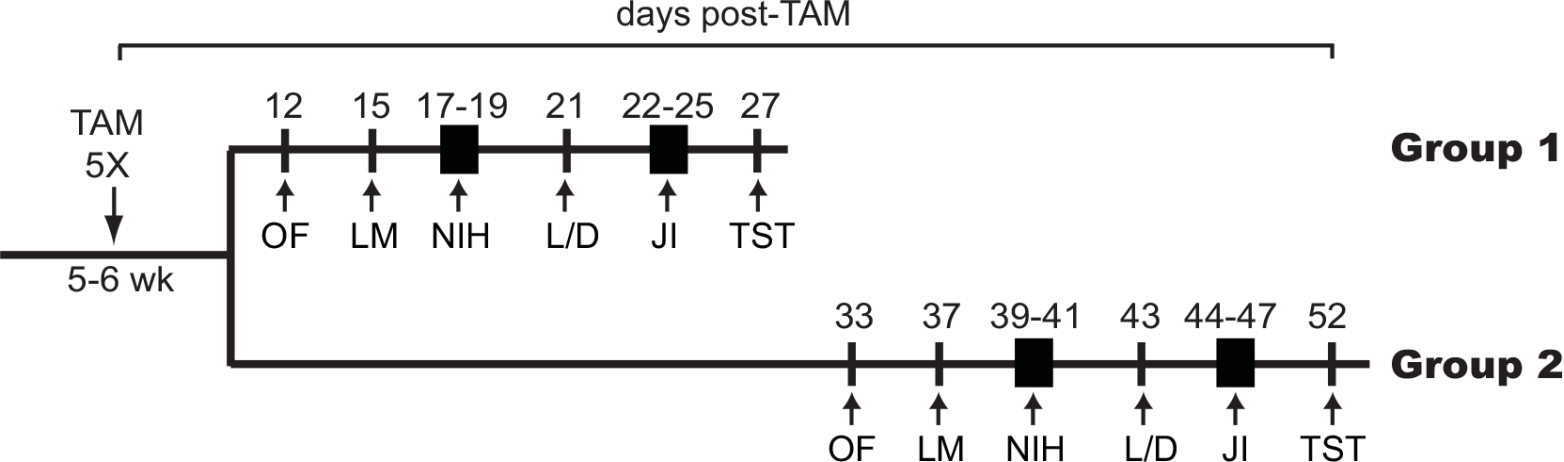
Experimental design of behavioral study. TAM was administered to 5–6 week-old control or Cre+DTA+ mice for 5 consecutive days. Behavioral testing began 12d (Group 1) or 33d post-TAM (Group 2), and continued as indicated through day 27 (Group 1, TAM-behavioral [TAM-beh] interval less than 4 weeks) or day 52 (Group 2, TAM-beh interval more than 4 weeks) post-TAM. Both groups were examined in the open field test (OF), locomotor test (LM), novelty induced hypophagia (NIH), light/dark test (L/D), juvenile social interaction test (JI), and tail suspension test (TST). Specifically for Groups 1 and Groups 2, OF was performed 12d or 33d post-TAM, LM 15d or 37d post-TAM, NIH 17-19d or 39-41d post-TAM, L/D 21d or 43d post-TAM, JI 22-25d or 44-47d post-TAM, and TST 27d or 52d post-TAM.

Male and female transgenic Nestin-CreER^T2^/DTA mice (Cre+DTA+) and littermates controls were given TAM at sexual maturity (5–6 weeks of age), an age often targeted in inducible transgenic mouse models in order to study adult neurogenesis [45, 47, 48]. TAM was purchased from Sigma-Aldrich (Cat. #T5648), prepared in stock solution (30 mg/ml made in 10% EtOH in sunflower seed oil), and given to all mice once a day for 5 consecutive days (see **Figs 1 and 2**) as an intraperitoneal (i.p.) injection at a dose of 180 mg/kg/day (6 ml/kg/day of stock solution).

### Overview of experimental design

**Fig 3:**
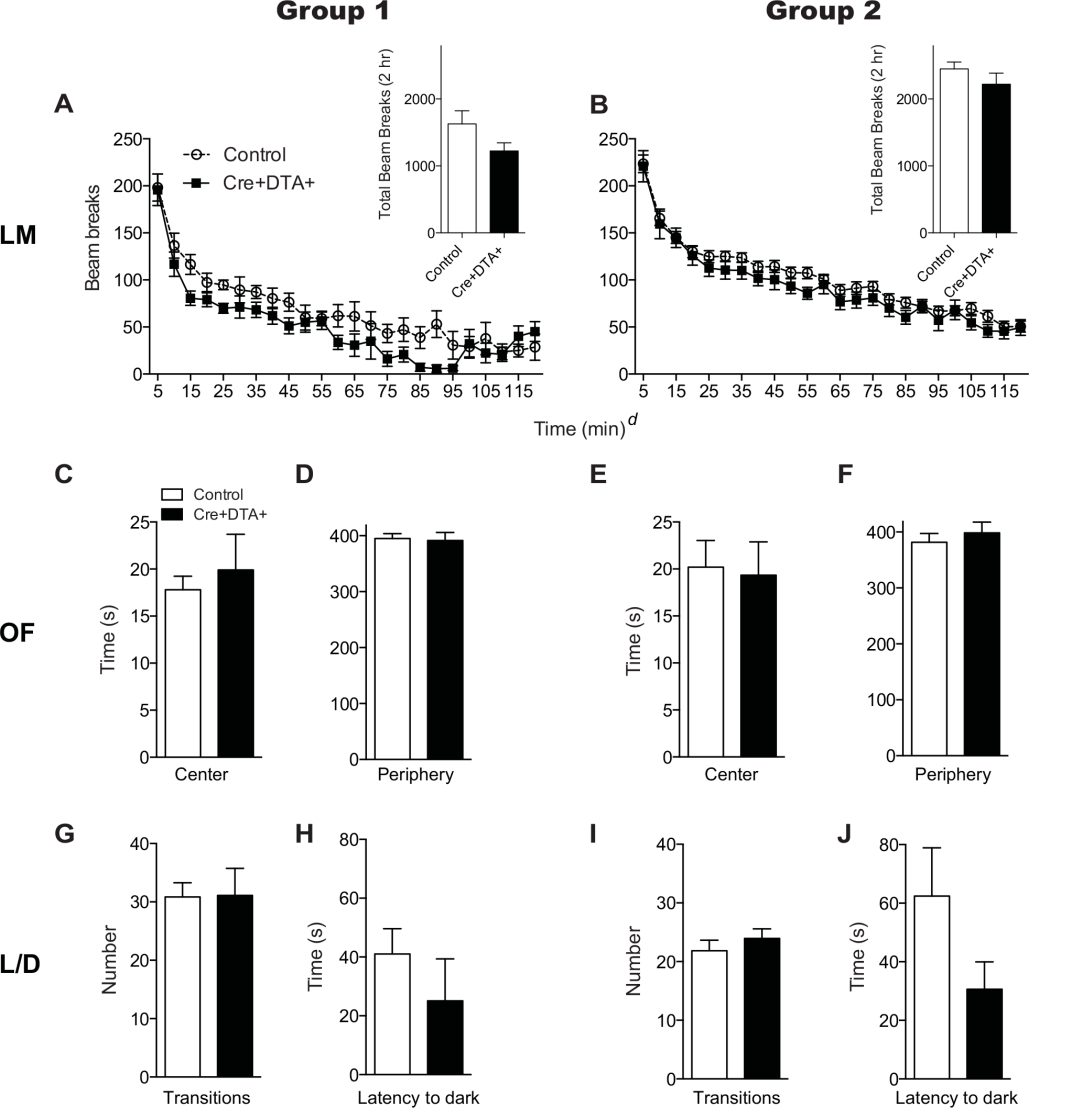
Cre+DTA+ mice tested less than or more than 4 weeks post-TAM show similar levels of locomotion and the absence of baseline anxiety-related behaviors. **(A, B)** Locomotor activity (LM) in both short (**A,** Group 1, control N = 8, Cre+DTA+ N = 5) and long (**B,** Group 2, control N = 33, Cre+DTA+ N = 13) TAM-beh interval groups. Insets **A, B**: total beam breaks over 2 hr. Main panels **A, B**: beam breaks over 2 hr presented in 5 min bins. X axis * = main effect of time. Posthoc analysis (Bonferroni) revealed all points in main panels were significantly different than the initial locomotor activity data point. However, individual data point asterisks are omitted for clarity, as there was no main effect of genotype or interaction of time X genotype for either Group 1 or Group 2. **(C-F)** Time spent in the center **(C, E)** and periphery **(D, F)** during an open field test (OF) in short (**C-D,** Group 1, control N = 30, Cre+DTA+ N = 9) and long (**E-F,** Group 2, control N = 42, Cre+DTA+ N = 17) TAM-beh interval groups. **(G-J)** Number of transitions between light and dark chambers **(G, I)** and latency to enter the dark chamber **(H, J)** in the light/dark test (L/D test) in both short (**G-H,** Group 1, control N = 30, Cre+DTA+ N = 9) and long (**H-I,** Group 2, control N = 38, Cre+DTA+ N = 17) TAM-beh groups. Data are mean±SEM. ^*d*^p<0.0001, two-way ANOVA with repeated measures and Bonferroni posthoc **(A, B)**. *p<0.05, unpaired two-tailed Student’s t-test (**insets A, B,** and **C-H**).

**Fig 4:**
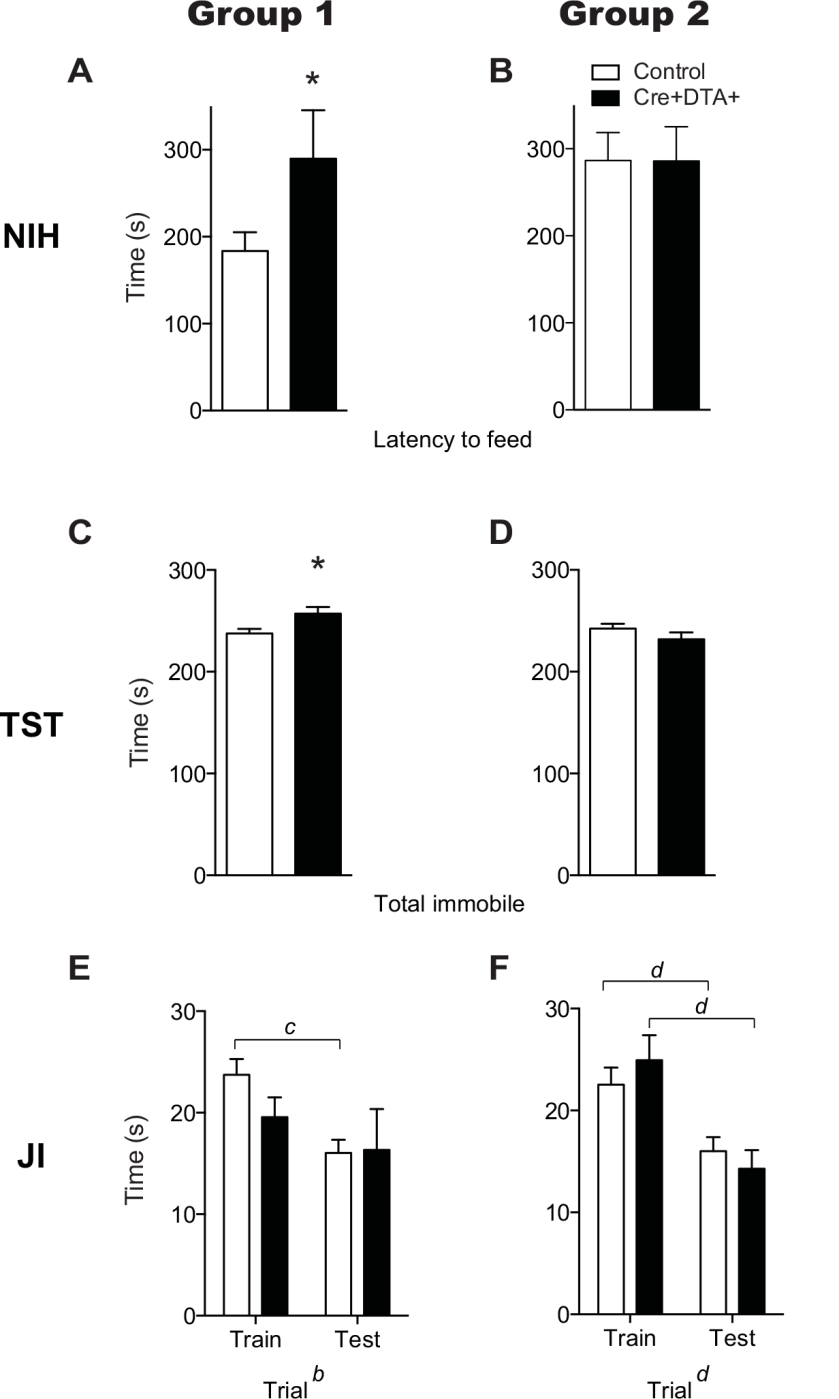
Relative to control mice, stress induced anxiety-like and depressive-like behavior are evident in Cre+DTA+ mice tested less than–but not more than– 4 weeks post-TAM. **(A, B)** Latency to feed in the novelty induced hypophagia test (NIH) in control vs. Cre+DTA+ mice at short (**A,** Group 1, control N = 31, Cre+DTA+ N = 10) and long (**B,** Group 2, control N = 39, Cre+DTA+ N = 16) TAM-behavior intervals. **(C, D)** Total immobility time in the tail suspension test (TST) in control vs. Cre+DTA+ mice at short (**C,** Group 1, control N = 27, Cre+DTA+ N = 9) and long (**D,** Group 2, control N = 38, Cre+DTA+ N = 15) TAM-behavior intervals. **(E, F)** Interaction time during juvenile interaction training and test sessions in control vs. Cre+DTA+ mice at short (**E,** Group 1, control N = 30, Cre+DTA+ N = 9) and long (**F,** Group 2, control N = 40, Cre+DTA+ N = 17) TAM-beh intervals. Data = mean±SEM. *p<0.05, unpaired two-tailed Student’s t-test (A-D). ^*b*^p<0.01,^*c*^p<0.005, ^*d*^p<0.0001, two-way ANOVA with repeated measures and Bonferroni posthoc.

Behavioral testing can influence neurogenesis [49–51]. Therefore, parallel groups of Cre+DTA+ and control mice treated for TAM were used for cellular analysis of neurogenesis (**Fig 1, S1 Fig**) and behavior (**Figs 2–4, S2 Fig**). Subject numbers (Ns) are provided in the Fig legends for each neurogenesis analysis or behavioral test and each time point. Neurogenesis data from Cre+DTA+ and Cre+DTA- mice are presented here, since other mice in the litter lacked the Cre gene and therefore would not express YFP, as was critical for the data collection in **S1 Fig.** Behavioral data from all littermates are presented here, as Cre- mice had behavior indistinguishable from Cre+DTA- mice. For behavior tests, control N were typically 3X greater than Cre+DTA+ mice due to our breeding strategy resulting in only 25% of the litter being Cre+DTA+, with the remaining 75% of the litter used for control.

### Tissue collection and sectioning

For neurogenesis analysis, TAM-treated Cre+DTA+ and control (Cre+DTA-) mice underwent transcardial perfusion and brains were collected at the time points indicated in **Fig 1**. Briefly, mice were anesthetized with chloral hydrate (Sigma-Aldrich cat. #C8383, 400 mg/kg, stock solution 400 mg/ml made in 0.9% NaCl solution, i.p.) and perfused intracardially with 0.1M PBS (7 ml/min, 6 min) and followed by perfusion in 4% paraformaldehyde in 0.1M PBS (7 ml/min, 15 min). As stress can influence neurogenesis, steps were taken to minimize potential stress differences among mice in the same cage: each cage was gently removed from the housing room and brought to the adjacent procedure room immediately prior to anesthesia; mouse cage transfer was performed by a researcher with clean personal protective equipment; and all mice in a cage were anesthetized within 3 min, and began exsanguination within 5 min, of being brought into the procedure room. With these and other steps, we have found neurogenesis levels in the Nestin-CreER^T2^ mouse line can be reliably and accurately evaluated with ~4 mice/treatment group, similar to N published in other neurogenesis studies [52–54], even when DG neurogenesis is quantified in only 1 hemisphere.

After perfusion, brain extraction, and subsequent cryoprotection, brains were bisected through the midsagittal sulcus, and one hemisphere was sectioned coronally on a freezing microtome (Leica). Thirty μm sections were collected through the entire anterior-posterior length of the hippocampus and DG (distance range from Bregma: -0.82 to -4.24 μm). As the mouse hippocampus is ~3.4mm long, and as 30um (0.03mm) coronal sections were collected in 9 serial wells, this means ~113 30um sections were collected through each hippocampus, with ~12 sections per well. Care was taken to “bookend” the hippocampus (including sections before and after the hippocampus in each well) to enable subsequent stereological quantification of neurogenesis.

### Immunohistochemistry (IHC)

Nine serial sets of sections were stored in 0.1% NaN3 in 1XPBS at 4°C until processing for slide-mounted IHC. For staining of Ki67+, DCX+, and YFP+ cells, one series of sections (e.g. 1 well containing every 9th section [~12 sections/well] through the hippocampus, sections in well 270um apart) were mounted on glass slides (Superfrost/Plus, Fisher) that were coded to ensure experimental remained blind throughout stereological analysis. Mounted sections were processed for antigen retrieval (0.01 M citric acid, pH 6.0, 95°C, 15 min), and nonspecific staining was blocked by incubation in blocking solution (3% normal donkey serum [NDS] vol/vol in 0.1% Triton X-100 in 1X PBS) for 30 min. Sections were then incubated in their respective primary antibodies: rabbit anti-Ki67 (1:500; Thermo Scientific, Cat. #RM-9106-S), goat anti-DCX (1:500; Santa Cruz, Cat. #SC-8066), or rabbit anti-GFP (1:3000; Invitrogen, Cat. # A11122) in 0.1% Tween-20 in 1XPBS overnight. The following day, sections were rinsed and incubated in biotinylated-donkey anti-rabbit IgG (1:200; Jackson ImmunoResearch, Cat. #711-065-162) or donkey anti-goat IgG antibody (1:200; Jackson ImmunoResearch, Cat. #705-065-003) in 1.5% NDS in 1XPBS for 1 hr. After rinses and 30 min incubation in 0.3% hydrogen peroxide in 1XPBS, and sections were incubated in avidin-biotin complex (Vector Laboratories) for 60 min. After rinsing, staining was visualized using DAB/metal concentration (Thermo Scientific, Cat. #1856090) or fluorescein-labeled tyramide signal amplication (PerkinElmer, Cat. #SAT701). Nuclear Fast Red (Vector Laboratories, Cat. #H-3403) or DAPI (Roche Cat. # 236276) was used as a counterstain.

### Stereological quantification and morphological categorization

Using an Olympus BX-51 microscope (Tokyo, Japan) and a 40X, 0.63 NA lens, cells immunoreactive for Ki67, DCX, and YFP cells in the subgranular zone (SGZ, Ki67+ cells), granular cell layer (GCL, DCX+ cells), or SGZ/GCL (YFP+ cells) respectively, of one hemisphere of the hippocampal DG were quantified via stereology. As previously described [36, 55–57], we quantified these relatively rare populations of cells within the dentate gyrus subgranular zone with the formula:

Total population of cells=total cells counted x1/ssf x1/asf x1/hsf

where ssf is the section sampling fraction (1/9), asf is the area sampling fraction (1 for these rare populations of cells), hsf is the height sampling fraction (1 given the minimal effect edge artifacts have in counting soma <10um with ssf 1/9), and where we only counted one hemisphere. Thus the resulting formula was:

Total population of cells in one hemisphere=total cells counted x1/(1/9)x1/1x1/1

Data presented here are from total counts, which was calculated:

Total population of cells=(Total population of cells in one hemisphere)x2

For morphological characterization, post-mitotic DCX+ cells were determined by presence of an apical process directed towards and extending into the molecular layer [58, 59], while subtypes of YFP+ cells (Type-1, progenitors/immature neurons, mature neurons, **S1 Fig**) were classified based on previously-reported morphological characteristics [45, 60, 61].

### Behavioral tests

The battery of test was performed from the least to the most stressful, as previously described [62]. As shown in **Fig 2**, two groups of Cre+DTA+ and control mice were given TAM and tested on a battery of behaviors to assess exploration and general locomotion (open field [OF], locomotor [LM]), baseline and stress-induced anxiety like behaviors (OF, novelty induced hypophagia [NIH], light/dark [L/D]), social memory (juvenile interaction [JI]), and depressive like behavior (tail suspension test [TST]). Based on the neurogenesis data **(Fig 1, S1 Fig)**, Group 1 and Group 2 began behavioral testing 12d or 33d post-TAM, respectively, in order to target times when neurogenesis in Cre+DTA+ mice was low (Group 1, 12d post-TAM or a short TAM-behavior interval) or had recovered and was similar to control mice (Group 2, 30d post-TAM, or a long TAM-behavior interval). Behavioral testing occurred during the light cycle by experimenter blind to treatment group. All mice in each group went through the same behavior tests. However, when data points had to be removed (due to unanticipated, occasional issues with behavioral testing equipment, individual mouse behavior, human error, or identification of outlier status by statistical analysis), subject number for that behavioral test was decreased accordingly. A brief description of each test used follows:

#### Open Field

OF probes exploratory behavior in a novel unfamiliar open space. During testing, mice tend to adhere to the periphery (thigmotaxis) as a consequence of an underlying propensity to avoid the potentially dangerous open center area [63, 64]. For the OF test, a mouse was placed under dim light in an open field made from a white Plexiglas chamber (44×44×30.5 cm) that was illuminated by fluorescent lights above the field’s bare floor (730 lux at cage floor). Using Ethovision software (Noldus Information Technology), the following movements were reported from the photocell sensor system surrounding the OF during the 5 min test: time spent in the center (14x14 cm) of the open field, latency to enter the center, frequency to enter the center, and time spent in the peripheral zone (5 cm around arena perimeter).

#### Locomotion

LM was assessed after mice were individually placed in a mouse cage with fresh bedding, and cage was placed between photocells under red light. A computer-controlled photobeam activity system (San Diego Instruments) recorded total movement of mice in the XY plane, with photocell beam breaks recorded with 5 min bins for 2 hr.

#### Novelty Induced Hypophagia

The NIH test is an ethologically-relevant paradigm for assessing the influence that a novel environment has on a previously-learned motivational experience, such as consumption of highly palatable food [65]. This test has excellent pharmacologic and ethological validation [30, 66], does not involve overnight food restriction, and minimizes food neophobia via the repeated exposure to the highly palatable food in a familiar cage. A component of the NIH test particularly useful for the present study is the stressful component of individual placement of the mouse in a familiar setting (home cage) for “training” trials and in novel, unfamiliar setting with distinct textural cues (e.g. fresh cage, corncob bedding instead of woodchip bedding) for “testing” trials. This allows comparison of latency to consume the highly palatable food in a familiar vs. an unfamiliar and potentially anxiety-provoking cage. Here the NIH was performed as described previously [65]. To reduce food neophobia, highly palatable food (graham cracker crumbs, Honey Maid, Nabisco, East Hanover, NJ) was placed in a petri dish in the home cage 24 hr prior to the 3 consecutive days for a total of 10 trials (9 training trials, 1 testing trial). Days 1 and 2 were strictly training days, with 3 training trials on Day 1 (trials 1.1, 1.2, 1.3) and 3 training trials on Day 2 (trials 2.1, 2.2, 2.3). Day 3 consisted of 4 trials (trials 3.1, 3.2, 3.3, 3.4) with trial 3.3 being the “test” in the unfamiliar cage, and trials 3.1, 3.2, and 3.4 being performed in the familiar, home cage. Each day, mice were placed into behavioral procedure room, their cage food was removed, and then mice were weighed 1 hr prior to the session. For each training trial, a dish containing 10 g of graham cracker crumbs was placed in the corner of the home cage. Then, individually, the mice were placed back into the home cage and latency to commence consumption of the palatable snack was recorded (up to a maximum of 10 min) by an observer blind to treatment group. For the test trial (session 3.3), testing was performed in the novel, unfamiliar cage. Latency to consume food was measured in each of the 10 trials (9 in the home cage, 1 in the unfamiliar cage) over the three days NIH.

#### Light/Dark

The L/D is interpreted as a “naturalistic” conflict test between spontaneous behaviors to explore novel environments and an aversion to bright light, and can be a useful adjunct measure of anxiety-like behavior [67, 68]. The L/D test assays anxiety-related behaviors during a 10 min session in a two chambered light/dark exploration model. The apparatus consisted of a polypropylene cage (44x21x21cm) unequally divided (⅔ and ⅓) into two chambers by a wall with a small vestibule. The large chamber was open, transparent, and brightly-illuminated by two 20W fluorescent lights (1388 lux at cage floor), while the small chamber was closed, painted black, and dark. Initially, the mouse was placed in the brightly-lit side, and the transitions of the mouse between the two chambers was automatically detected by four photocells located in the vestibule. The latency to enter the dark side was measured, as was the number of transitions between the light and dark sides. Many control mice (N = 2 12d post-TAM, N = 9 30d post-TAM) had a long latency to enter the dark chamber, leading to a bimodal distribution of the data and potential consideration of these mice as outliers. In contrast, DTA mice were far less likely to have a long latency to enter the dark chamber (N = 2 12d post-TAM, N = 0 30d post-TAM), and thus DTA data notably lacked this bimodal distribution (scatterplots not shown). However, both control and DTA mice had similar distribution in other L/D measures (number of transitions) and measures in other behavioral tests, and the results for L/D was the same whether these outliers were kept in or omitted for L/D analysis. Therefore, the L/D data reported here are from all mice, regardless of bimodal distribution for the latency to enter the dark chamber.

#### Juvenile Social Interaction

Social memory can be tested in rodents via interaction assessment, such as placing a juvenile in a cage with an adult [69]. The adult will spontaneously investigate the younger animals and total interaction time indicates the familiarity between two animals. JI consisted of two days of behavioral analysis with one trial on each day (Day 1 trial was training, Day 4 trial was testing). Mice in home cage were placed in the testing room in the dark under red light for 15 min prior to Day 1 and Day 4. The 2 min training trial on Day 1 consisted of placing an individual mouse into a clean, unfamiliar mouse cage with no bedding along with an unfamiliar 4-week-old mouse of the same sex. Three days later, on Day 4, a 2 min testing trial was performed with the same procedure as the training trial and same juvenile mouse [70, 71]. Time spent interacting (following, sniffing, etc.) with the juvenile during both the training trial on Day 1 and testing trial on Day 4 were measured by an observer blind to treatment.

#### Tail Suspension Test

The TST measures the stress response or “behavioral despair”, and it a common behavioral task relevant to depression as it has good pharmacologic validity [72–74]. The TST allows assessment of behavioral despair in mice exposed to an inescapable situation that manifests as immobility after a period of struggling. The test used an automated TST device (Med Associates) where a mouse was suspended by the tail using tape (Scotch Super Strength mailing tape Cat. #341, 3M Corp, St. Paul, MN) to an aluminum bar connected to a strain gauge. The strain gauge detected movements of the mouse and transmitted them to a central unit. The total duration of immobility was automatically calculated as the time the force of the mouse’s movements was below a pre-set threshold criterion (i.e. immobile and not struggling) during the 6 min TST test. The device’s force transducer used pre-set thresholds to determine time spent immobile (behavioral withdrawal) and time spent struggling (escape-oriented behavior) with the following settings: time constant = 0.25, gain = 4, threshold 1 = 3, and resolution = 200 msec. Whenever the mouse’s movements were lower than our threshold 1 (<3) for 200 msec, then the duration of the immobility accumulated. The duration of immobility were detected and calculated by the computer [72]. The data from mice that exhibited excessive climbing behavior during the test were omitted from the short TAM-behavior interval group (3 control) and long TAM-behavior interval group (4 control, 2 Cre+DTA+).

### Statistical Analyses

Statistical analyses were performed using Prism (GraphPad vs. 6.0) or SPSS software (V19). Analyses on data with two groups (total Ki67+, DCX+, and YFP+ total cell number, and OF, LM total beam breaks, TST, L/D) were performed using an unpaired two-tailed Student’s t-test. Analyses on data with more than two variables (YFP+ cell phenotype between genotypes, novelty induced hypophagia trials each day between genotypes) were performed with two-way ANOVA with Bonferroni posthoc test for multiple comparisons. Analyses on data with more than two variables that required repeated measures (locomotor test in 5 min bins between genotypes, JI trials) were performed with two-way ANOVA with repeated measures (time or trial, respectively), with Bonferroni posthoc test for multiple comparisons. Statistical significance was defined as p<0.05, and threshold significance value is presented (e.g. p<0.05, p>0.05, p<0.0001, etc.). Outliers on specific tests (e.g. TST) were determined via mouse observation (climbing) or SPSS, and these values were removed and the N for the group was adjusted to reflect this. However, if analysis revealed a bimodal distribution (e.g. L/D), outliers were not omitted, and instead the bimodality is noted in the appropriate section.

## Results

### Nestin-CreER^T2^/DTA transgenic mice have a transient reduction in DCX+ neuroblasts/immature neurons and YFP+ cells, but a long-lasting reduction in Ki67+ progenitors

To inducibly ablate nestin-lineage stem/progenitor cells in the adult mouse, Cre+DTA+ and control mice were given TAM (**Fig 1A**). As previously described with this nestin-CreER^T2^ driver [45], TAM administration induces CreER^T2^ translocation into the nucleus of nestin-expressing cells. By combining with the DTA mouse, this allows expression of DTA in nestin-expressing neural stem/progenitor cells (NSCs) [44]. Quantification of progenitors (Ki67+ cells, **Fig 1B and 1C**) and neuroblasts/immature neurons (DCX+ cells, **Fig 1D and 1E**) was performed 12d and 30d post-TAM in Cre+DTA+ and control mice. Cre+DTA+ mice had 53% fewer total Ki67+ SGZ cells than control mice 12d post-TAM (p<0.01, **Fig 1C**), and 25% fewer Ki67+ SGZ cells 30d post-TAM (p<0.05, **Fig 1C**). Cre+DTA+ mice also had 18% fewer DCX+ GCL cells compared to control mice 12d post-TAM (p<0.05, **Fig 1E**). However, Cre+DTA+ and control mice had similar number of DCX+ GCL cells 30d post-TAM (p>0.05, **Fig 1E**). This transient decrease in total DCX+ cells was also apparent when just postmitotic DCX+ cells were quantified (**Fig 1F and 1G**; [58, 59]), as Cre+DTA+ mice had 28% fewer postmitotic DCX+ cells than control mice 12d post-TAM (p<0.05), but similar number of postmitotic DCX+ cells as control mice 30d post-TAM (p>0.05, **Fig 1G**). These significant decreases in proliferation (Ki67+ cells) and neurogenesis (DCX+ postmitotic cells) 12d post-TAM were further supported by a decrease in total YFP+ cells (55%), YFP+ Type-1 cells (54%), and YFP+ progenitors/immature neurons (55%) 12d post-TAM **(S1A–S1E Fig**), but no change in total YFP+ cells or any YFP+ cell phenotypes 30d post-TAM **(S1F Fig)**. These data show inducible expression of the DTA transgene in nestin-lineage cells results in reduction in progenitors and neuroblasts/immature neurons 12d post-TAM, but that the reduction in neuroblasts/immature neurons is transient since it is normalized by 30d post-TAM.

### Transient TAM-induced reduction in DCX+ and cell number does not change total locomotion activity and does not result in baseline anxiety-like behavior

Having established that Cre+DTA+ mice have a transient reduction in neuroblasts/immature neurons relative to control mice, we examined new cohorts of mice to examine whether transiently-reduced neurogenesis led to changes in general behavior (locomotion, baseline anxiety-like behavior, **Fig 2**) or behavior relevant to stress-induced anxiety, depression, or social memory.

Cre+DTA+ and control Group 1 mice (examined at a short TAM-behavior interval) had no difference in locomotor activity when examined as total beam breaks during the 2 hr test (p>0.05 **Fig 3A inset**). Analysis of locomotor activity as 5 min bins (**Fig 3A main panel**) revealed significant effects of time (two-way ANOVA, F_23,253_ = 36.57, p<0.0001) and subject (matching; F_11,253_ = 16.84, p<0.0001) but no main effect of genotype (F_1,11_ = 2.266, p>0.05) or time X genotype interaction (F_23,253_ = 1.344, p>0.05). Posthoc analyses revealed the expected significant decrease in activity over time, with all activity significantly lower than the initial locomotor activity (p’s<0.05). Similar effects were seen in Cre+DTA+ and control Group 2 mice (examined at a longer TAM-behavior interval): no difference in locomotor activity when examined as total beam breaks over 2 hr test (p>0.05, **Fig 3B inset**), but data plotted over time (**Fig 3B main panel**) revealing main effects of time (F_23,1012_ = 92.40, p<0.0001) and subject (matching; F_44,1012_ = 22.51, p<0.0001), but no main effect of genotype (F_1,44_ = 1.387, p>0.05) or time X genotype interaction (F_23,1012_ = 0.5397, p>0.05). Again posthoc analyses revealed the expected significant decrease in activity over time, with all activity significantly lower than the initial locomotor activity (p’s<0.05). These locomotor data from Group 1 and Group 2 support that subsequent behavior results would not be grossly compromised by different locomotor activity or habituation between Cre+DTA+ and control mice, even when examined at the relatively short time period post-TAM when progenitors and neuroblasts/immature neurons were reduced in Cre+DTA+ mice (**Fig 1**).

We next examined the performance of Cre+DTA+ and control mice in Group 1 and Group 2 on OF **(Fig 3C–3F)** and L/D **(Fig 3G–3J)** to assess anxiety-like behaviors. Irrespective of whether mice were examined at a short TAM-behavior interval (Group 1) or a long TAM-behavior interval (Group 2), there was no difference between Cre+DTA+ and control mice in time spent in the center (Group 1 p>0.05, **Fig 3C**; Group 2 p>0.05, **Fig 3E**) or periphery (Group 1 p>0.05, **Fig 3D**; Group 2 p>0.05, **Fig 3F**). There was a similar lack of effect in L/D (**Fig 3G–3J**). Irrespective of whether mice were examined at a short TAM-behavior interval (Group 1) or a long TAM-behavior interval (Group 2), there was no significant difference between Cre+DTA+ and control mice in number of transitions between chambers (Group 1 p>0.05, **Fig 3G**; Group 2 p>0.05, **Fig 3I**) or latency to enter the dark chamber (Group 1 p>0.05, **Fig 3H**; Group 2 p>0.05, **Fig 3J**). These data suggest DTA-dependent transient reduction in neurogenesis does not alter exploratory or baseline anxiety-like behavior. This agrees with prior work showing no correlation between genetically-ablated adult neurogenesis and anxiety behavior when examined at baseline [31, 32, 34].

### Transient TAM-induced reduction in DCX+ cell number results in stress-induced anxiety and depressive-like behavior, but no change in a test of social memory

Having established that DTA-dependent transient reduction in neurogenesis does not alter general exploratory or baseline anxiety-like behavior, we considered the performance of Group 1 and Group 2 Cre+DTA+ and control mice on behavioral tests that offer insight into stress-induced anxiety, depression-like behaviors, and social memory: the NIH, TST, and JI tasks, respectively. In the NIH test, Group 1 Cre+DTA+ mice (examined at a short TAM-behavior interval) took 42% longer to consume highly palatable food in the unfamiliar cage, but not in the familiar cage as compared to control mice **(Fig 4A**, Group 1 p<0.05; **S2 Fig**, Group 1 main effects of trial [F_3,156_ = 10.14, p<0.0001] and genotype [F_1,156_ = 4.752, p<0.05], but no trial X genotype interaction [F_3,156_ = 0.9536, p>0.05]). However, at a longer TAM-behavior interval, Group 2 Cre+DTA+ and control mice showed similar latency to consume the highly palatable food (**Fig 4B,** Group 2 p>0.05; **S2 Fig**, main effect of trial [F_3, 208_ = 8.058, p<0.0001] but no main effect of genotype [F_1, 208_ = 0.1038, p>0.05], and no trial X genotype interaction [F_3, 208_ = 0.1495, p>0.05]). Thus, transient TAM-induced reduction in DCX+ cell number results in increased latency to consume the highly palatable food in an unfamiliar cage but not in a familiar cage, suggesting the deficit is seen under stressful or anxiety-provoking conditions.

To complement the testing of stress-induced anxiety, mice were also examined in the TST In the TST, Cre+DTA+ mice examined at a short TAM-behavior interval showed significantly more total immobile time compared to control mice (Group 1 p<0.05, **Fig 4C**). However, as with the NIH test, this effect was transient, as at a longer TAM-behavior interval Cre+DTA+ mice and control mice showed similar immobile time (Group 2 p>0.05, **Fig 4D**). Thus, coupled with the lack of DTA-dependent difference in other anxiety tests (**Fig 3C–3J**), these NIH and TST data suggest transient reduction in progenitors and neuroblasts/immature neurons is associated with stress-induced anxiety and depressive-like behavior.

A variety of neuropsychiatric disorders including depression are characterized by disruptions in social behavior and social recognition [75]. Since Cre+DTA+ mice examined at a short TAM-behavior interval showed depressive-like behavior, we also examined whether these mice were different from controls in social interaction or social memory. Analysis of juvenile interaction in Group 1 mice (short TAM-behavior interval; **Fig 4E**) revealed main effects of trial (two-way ANOVA, F_1,37_ = 8.345, p<0.01) and subject (matching; F_37,37_ = 1.747, p<0.05) but no main effect of genotype (F_1,37_ = 0.6022, p>0.05) or trial X genotype interaction (F_1.37_ = 1.403, p>0.05). Posthoc analyses of trial via Bonferroni revealed a significant lower interaction time in test trial vs. train trial in control mice (p<0.001) not in Cre+DTA+ mice. Analysis of juvenile interaction in Group 2 mice (long TAM-behavior interval; **Fig 4F**) revealed main effects of trial (two-way ANOVA, F_1,55_ = 43.66, p<0.0001) and subject (matching; F_55,55_ = 3.493, p<0.0001) but no main effect of genotype (F_1,55_ = 0.0245, p>0.05) or trial X genotype interaction (F_1,55_ = 2.516, p>0.05). Posthoc analyses of trial via Bonferroni revealed a lower interaction time in test trial vs. train trial in both control mice and Cre+DTA+ mice (p’s<0.0005). Taken together, these NIH, TST, and JI data suggest transient reduction in progenitors and neuroblasts/immature neurons is associated with indices of stress-induced anxiety and depressive-like behavior but no robust change in a test of social memory.

## Discussion

While much has been written about the “neurogenic hypothesis of affective and anxiety disorders”, it remains unclear whether the number of new neurons in the adult DG relates to depression and anxiety, and the expression of depressive-like behaviors. For example, some studies find that inducible reduction of DG neurogenesis results in baseline anxiety and depressive-like behaviors, but many do not [21]. The reasons for these conflicting data have been discussed extensively elsewhere [28, 29, 76, 77], and include the stress state of the animal, the mode and efficiency of ablation, the interval between ablation and behavioral testing, and the behavioral tests employed [21]. Here we show that induced, transgenic-mediated, transient reduction in DCX+ neuroblasts/immature neurons resulted in stress-induced anxiety and depressive-like behavior. While Ki67+, postmitotic DCX+, and YFP+ progenitors and immature neurons were reduced by ~50%, 30%, and 60%, respectively, at the early time point post-TAM when the mice demonstrated stress induced anxiety and depressive-like behavior, DCX+ and YFP+ cell numbers were normalized at the later time points post-TAM when the stress-induced anxiety and depressive-like behavior was not detectable. These results show that induced, transient reduction of DCX+ cell number is linked to a component of affective disorders. Below we discuss these results in relationship to the dynamics of neurogenesis and the published literature on the controversial relationship between neurogenesis and mood disorders. We also discuss whether or not our results support a stage-specific role for adult born DG neurons in depressive- and anxiety-like behavior.

### Transient reduction in neuroblasts/immature neurons after ablation: relationship to depressive-like behavior?

Many studies show that viral mediated- or transgenic-induced decreases in neurogenesis recover over time [37, 39, 78, 79]. In contrast, irradiation-induced decreases in neurogenesis do not recover, presumably due to the more severe disruption of the components of the neurogenic niche, destroying both “seed and soil” [24, 40, 80]. This variability in neurogenesis regeneration can be exploited to investigate the differential contribution of neurogenesis to behavior and physiology. Recently an inducible transgenic mouse with transient reduction in surviving adult-generated neurons showed no depression-related behaviors [30]. However, the surviving adult-generated neurons that were the focus of that study are only one functional component of the process of neurogenesis. Cells in “earlier” stages of neurogenesis, like progenitors and neuroblasts/immature neurons, are known to serve both neurogenic and nonneurogenic functions in the adult DG [33, 42]. Here, our inducible transgenic mouse with transient reduction in DCX+ cells showed depression-related behaviors. Considered along with Deng et al., 2015, our present data suggest “stage”-specific roles for adult-generated cells in depressive-like behavior.

These studies join a host of others that suggest “stage”-specific roles for adult-generated cells–particularly DCX+ cells–in other, non-depression related hippocampal-dependent behaviors. For example, transient depletion of DCX+ cells (~80%) in a DCX-diphtheria toxin receptor (DTR) knock-in mouse results in impaired learning of place avoidance shortly after DT administration [41]. However, when DCX+ cell number recovered 1 month later, spatial learning behavior was normalized. Notably, reduced neurogenesis and learning correlated with reduced number of DCX+Arc+ (**a**ctivity-**r**egulated **c**ytoskeleton-associated protein) cells as well with reduced total Arc+ DG cells during learning. This suggests that a reduction in DCX+ cell number contributes to decreased DG activity during learning. Interestingly, stress and antidepressants have the opposite effect on the DG activity. For example, chronic mild stress (CMS) leads to decreased ventral DG activity, while chronic antidepressant treatment leads to increased ventral DG activity [35]. Adult hippocampal neurogenesis appears to play a role in both the antidepressant efficacy and the subsequent antidepressant-induced enhancement in DG activity [16, 35, 81]. As a whole, these studies support the hypothesis that transient reduction of immature neurons–particularly by physiological stimuli like stress [24, 32, 82, 83]–leads to decreased DG activity and depressive-like behavior, and cellular recovery is associated with normalized DG activity and behavior [25, 35, 41, 81].

Notably, there are other inducible, transient tools that could be employed for future testing of this hypothesis [30, 37, 84, 85]. For example, transgenic-induced reduction in neurogenesis in the nestin-inducible Bax bigenic mouse that overexpressing Bax recovers 4 weeks later, and this recovery is accompanied by normalization of key electrophysiological characteristics of the hippocampus [84]. This nestin-inducible Bax mouse has also been tested for mood-related behaviors, but only when neurogenesis was reduced. Interestingly, ablation of neurogenesis led to baseline anxiety-like, but not depressive-like, behaviors. As our data here suggest that the behavioral profile of mice changes along with recovery of neurogenesis, it would be very interesting to test how the behavioral profile of this nestin-inducible Bax mouse—and other models of transient reductions in neurogenesis—changes over time, as such data will significantly advance efforts to dissect a potential temporal relationship between neurogenesis and affective behaviors.

### Neurogenesis and anxiety-like behavior

Anxiety and major depressive disorders are often comorbid in humans. However, as with depression and neurogenesis, the relationship with anxiety and neurogenesis is unclear. For example, in rodent studies running on an exercise wheel is anxiogenic [86] and also increases neurogenesis [51]. Moreover, neurogenesis ablation model using nestin-inducible TK transgenic mouse fails to show baseline anxiety-like behaviors [30]. Paradoxically, neurogenesis ablation in the nestin-inducible Bax transgenic mouse increases baseline anxiety-related behaviors [17]. However, there is not a straightforward relationship between new neuron number and anxiety, since both running-induced enhancement in adult neurogenesis and ablation of the running-induced enhancement are anxiogenic [86, 87]. Also, many neurogenesis ablation models fail to show baseline anxiety-like behaviors in EPM, OF, L/D or other tests [30, 31, 34, 87, 88]. Taken together, this mixed literature emphasizes the importance of looking over time and assessing both baseline and stress induced anxiety- and depression-like behaviors.

Here we assessed both baseline and stress induced anxiety-like behaviors and depressive-like behavior in our mouse model of transiently-disrupted neurogenesis. While our Cre+DTA+ mice have transiently fewer DCX+ cell number and display depressive-like behaviors, they do not display baseline anxiety-like behavior in the L/D, OF, or EPM tests (data not shown) at any time point examined. Our results are consistent with results from other neurogenesis ablation models, such as the GFAP-TK mouse line [34], and also with a key message that emerged from a meta-analysis of the field [89]: ablated neurogenesis leads to depressive-, but not baseline anxiety-like, behaviors.

What might explain the diverse findings in the literature between depression-like and anxiety-like behaviors and neurogenesis? One concept, formally advanced by Snyder et al., in 2011, is that new neurons buffer the stress response [34], and therefore loss of new neurons would be functional or evident primarily in times of stress. In our transient TAM-induced reduction in DCX+ cell number results in only stress-induced anxiety in the NIH not baseline anxiety tests. Recently, this concept was tested under gain-of-neurogenesis conditions [38]. Induced-deletion of Bax in nestin-expressing cells increased hippocampal neurogenesis and had no influence on baseline anxiety and depressive-like behaviors. In contrast, inducible increased hippocampal neurogenesis blunted anxiety- and depressive-like behaviors produced in a mouse model of stress [20, 38, 90, 91]. Clearly, additional studies are warranted to further test the hypothesis that neurogenesis differentially affects mood-related behavior under baseline conditions and after stress. Based on our data presented here, we propose such future studies utilize transient reductions in neurogenesis if possible, and also consider the role for DCX+ cell number as well as more mature cells in the resulting behavioral performance.

### Future directions

Given the specificity of the genetic components of the bigenic mouse model used here (e.g. Cre-induced recombination is only evident in nestin-lineage cells [45, 92]), our results suggest that the transient display of stress-induced anxiety and depression-like behavior is due to transient decrease in neuroblasts and immature neurons. However, future work is needed to make a firm conclusion, and to test the associated hypothesis that immature neurons are functionally important in mood-related behaviors.

For example, the field continues to be limited by a lack of consistent definition of what constitutes depressed or anxious behavior in animal models, and specifically what defines “stress-induced behavior” [93]. Certainly, experimentally-induced “despair” or “anxiety” are commonly measured using a battery of tests, as is performed in the present work. However, these phenotypes are induced in a variety of ways, from acute stress to repeated injection of a stress hormone, such as corticosterone, and the resulting phenotype does not always respond to antidepressant or antianxiolytic interventions [28, 76]. In addition, given that animal models typically show a heterogeneous response in a battery of tests—as in the present work, where there is no obvious anxiety phenotype but significant effects in the novelty induced hypophagia and tail suspension test—the declaration of a resulting phenotype as “depressive-like” is subjective rather than objective. One way to address this challenge is to utilize more standardized approaches to inducing depression, such as repeated application of uncontrollable or unpredictable stress, and importantly to couple this induction with quantifiable measures of stress hormones (e.g. corticosterone), stress and stress hormone-sensitive measures (e.g. weight change, adrenal hypertrophy), and behavior (e.g. grooming, eating, or social interaction) [28, 93, 94]. While we believe it is reasonable to interpret our data as suggesting a functional association between adult neurogenesis and stress induced anxiety- and depressive-like behaviors, future studies are warranted to test this association under conditions that clearly and physiologically mimic stress. We would predict that depression-like behavior in our mouse line would be amplified when tested in the context of, for example, social defeat stress [36, 95].

Also, use of alternative approaches to transiently reduce new neurons will allow mechanistic dissection of our conclusion. For example, is depressive-like behavior driven by loss of a neurogenic, non-neurogenic function, or both functions of immature neurons? Such questions could be addressed via chemogenetic-induced silencing of cells over a period of time. Another set of future studies are prompted by the design of our experiment, in that our conclusion relies on static measurement of neurogenesis indices at two time points post-TAM as well as dynamic, ongoing measurements of behavior starting at short or longer time points post-TAM. While we specifically did not examine neurogenesis in mice that went through behavioral testing (as behavior testing is well-documented to itself change neurogenesis), future studies monitoring neurogenesis and DG functionality in awake and behaving animals are warranted [96].

Another important consideration is whether the relatively small decrease in DCX+ cells reported here can actually be responsible for a behavioral change. In this regard, it is useful to note that we find postmitotic DCX+ cells and YFP+ (e.g. nestin-lineage cells) progenitors/immature neurons decreased to a larger extent (30% and 60%, respectively) than total DCX+ cells (20%). Decreased neurogenesis in this range of magnitude certainly has been shown to result in behavioral change [30, 31, 34, 37, 97, 98]. This underscores both the integration of postmitotic cells into DG-CA3 circuitry [13, 99–102], and that loss of these cells may indeed influence behavior. Certainly, the nature of DG-CA3 synapse supports a non-linear relationship between cell number and behavior, as one DG GCL neuron can have significant impact on downstream structures despite representing a small fraction of the DG GCL neuron population [103, 104]. However, as we did not rescue neurogenesis in our model, but rather let it recover over time, it is not yet possible to attribute the behavioral change we report specifically to a loss of neurogenesis. In fact, it is also useful to note that inducible gene deletion in a specific lineage of cells can result in a compensatory response outside that lineage. We have recently referred to this as a “community level” response, where inducible loss of new neurons in one lineage has a secondary or cell non-autonomous effect on cells outside of that lineage [58]. As it is currently unclear whether the change in behavior reported here is due to the transient loss of neurogenesis, to some secondary effect, or both, here we opted to report several measures of decreased neurogenesis, including the decrease in lineage-specific cell number (YFP+ cells) as well as total and postmitotic DCX+ cell number. We hope providing these additional measures will help advance our understanding of whether the magnitude of decrease or the lineage of cells directly affected relate to the behavioral outcome. The relationship among the magnitude of cell loss, the lineage influenced, the community level response, and resulting behavioral effects (or lack thereof) is proving to be complex, and thus warrants additional studies. In this regard, well-designed studies that have negative results are also of great interest to the field [105–109], and it is hoped that researchers will recognize the importance of having negative data in general published.

Finally, and on a related note, it may be that there is not a direct relationship between reduction in neuroblast cells and the behaviors reported here, and that other intermediates are ultimately producing our behavioral changes. For example, changes in glia, inflammatory signals, or vasculature can augment the depressive-like phenotype [18, 33, 110, 111]. While other ablation methods have side effects (i.e. nestin-TK or irradiation leading to vascular changes or impaired cellular function of other brain regions) [30, 40, 80], the DTA line that we used here has not had any obvious side effects reported [44]. Therefore it will be important to carefully assess whether indices of glia, inflammatory signals, or vasculature markers are evident after TAM in this Nestin-CreER^T2^/DTA line.

## Conclusions

The neurogenesis hypothesis of depression and anxiety is supported by many correlative findings (e.g. postmortem analysis of brains from humans diagnosed with MDD, or animals given chronic antidepressants) and an increasing number of causative studies (e.g. irradiation, antimitotic agents) [21]. More recently, studies using transgenic-mediated ablation of neurogenesis have provided more insight into whether and how new neurons relate to the development and treatment of affective and anxiety disorders [34, 38], and these add to the growing complexity and intrigue of the research findings, such as the apparent importance of new neurons particularly during times of stress. This complexity is likely a main reason that a clear relationship among neurogenesis, stress, and affective disorders has yet to emerge, even after the publication of many studies. However, this complexity is also precisely the reason to call for more, not fewer, well designed and controlled studies to probe and understand the complete cascade of steps and mechanisms underlying the potential association between neurogenesis ablation and an anxiety- and depressive-like phenotype, and to test the hypothesis—not to prove the theory—that ablated neurogenesis leads to or exacerbates mood disorders.

Our findings highlight that early neurogenic cells are functionally associated with stress-induced anxiety and depressive-like behavior in adult mice. The concept that young adult-generated cells contribute to behaviors relevant to mood regulation is novel, and these findings therefore offer a unique perspective on etiology of anxiety and depression. Also, our data underscore that the timing of the behavioral testing relative to ablation is crucial. While this is not surprising since adult neurogenesis is a dynamic process, it is rare that the issue of timing is as obvious as presented here. Taken together, our work highlights the importance of additional study with other transient neurogenesis ablation approaches followed by the examination of anxiety- and depressive-like behaviors.

## Supporting Information

S1 Fig Inducible expression of DTA in nestin-lineage stem/progenitor YFP+ cells decreases the number of total YFP+ cells 12d, but not 30d, post-TAM, as well as decreases specific phenotypes of YFP+ cells, including Type-1 cells, progenitors, and immature neurons. **(A)** Representative photomicrograph of DG from a control mouse 30d post-TAM stained with an antibody against YFP (pink; blue, DAPI counterstain). **(B-D)** Representative photomicrographs of nestin-lineage YFP+ cells, including Type-1 radial glial-like neural stem cell **(B)**, progenitors/immature neurons **(C)**, and mature neurons **(D)**. **(E,F)** Stereological quantification of YFP+ cells in the DG GCL 12d (**E**, control N = 3, Cre+DTA+ N = 2) and 30d (**F,** control N = 3, Cre+DTA+ N = 3) post-TAM. Total YFP+ cell number was significantly decreased in Cre+DTA+ mice 12d post-TAM (**E**, p<0.05), but total YFP+ cell number was similar between control and Cre+DTA+ mice in 30d post-TAM group (**F**). Phenotypic analysis of YFP+ cell types via two-way ANOVA (variables genotype and phenotype) revealed at 12d post-TAM that there were main effects of genotype (F_1,9_ = 29.59, p<0.005) and phenotype (F_2,9_ = 62.54, p<0.0001), and a genotype X phenotype interaction (F_2,9_ = 9.632, p<0.01), and 30d post-TAM no main effects or interactions (p’s>0.05). Posthoc analysis on 12d post-TAM data revealed significantly fewer Type-1 cells and progenitors/immature neurons in Cre+DTA+ mice relative to control mice. Phenotypes posthoc not shown. *p<0.05, unpaired Student’s t-test; ^*a*^p<0.05, ^*b*^p<0.01, ^*c*^p<0.005, ^*d*^p<0.0001, two-way ANOVA with Bonferroni posthoc. Data represent mean±SEM. Scale bar = 200 um **(A)**, 20 um **(B)**.
(EPS)

S2 Fig NIH testing (Day 3) reveals significant main effects of trial and genotype in the 12d, but not 30d, post-TAM group. Mice underwent 2 training days with 3 trials per day (1.1, 1.2, 1.3, 2.1, 2.2, 2.3) at a short TAM-behavior interval (Group 1) or long TAM-behavior interval (Group 2). While not shown, two-way ANOVA of Day 1 and Day 2 Group 1 and Group 2 data revealed: Group 1, Day 1 main effect of trial (F_2,117_ = 27.51, p<0.0001), but no main effect of genotype (F_1,117_ = 3.320, p = 0.07) or trial X genotype interaction (F_2,117_ = 1.630, p>0.05); Group 1, Day 2 no main effect of trial (F_2,117_ = 1.862, p>0.05) or genotype (F_1,117_ = 0.5077, p>0.05), and no trial X genotype interaction (F_2,117_ = 0.2022, p>0.05); Group 2, Day 1 main effect of trial (F_2,156_ = 30.19, p<0.0001), but no main effect of genotype (F_1,156_ = 0.03665, p>0.05) or trial X genotype interaction (F_2,156_ = 0.2096, p>0.05); and Group 2, Day 2 no main effect of trial (F_2,156_ = 2.597, p = 0.077) or genotype (F_1,156_ = 0.2245, p>0.05), and no trial X genotype interaction (F_2,156_ = 0.07013, p>0.05). On Day 3, NIH mice were trained in the familiar home cage for three trials (3.1, 3.2, 3.4) and tested in an unfamiliar cage for one trial (3.3). **(A)** When Group 1 Day 3 trials were examined (short TAM-behavior interval) via two-way ANOVA (trial x genotype), there were main effects of trial (F_3.156_ = 10.14, p<0.0001) and genotype (F_1,156_ = 4.752, p<0.05), but no trial x genotype interaction (F_3,156_ = 0.9536, p>0.05). **(B)** When Group 2 Day 3 trials were examined (long TAM-behavior interval) via two-way ANOVA (trial x genotype), there was a main effect of trial (F_3,208_ = 8.058, p<0.0001), but not of genotype (F_1,208_ = 0.1038, p>0.05) and no interaction of trial x genotype (F_3,208_ = 0.1495, p>0.05). Trial posthoc not shown. ^*a*^p<0.05, ^*d*^p<0.0001, two-way ANOVA with Bonferroni posthoc. Data represent mean±SEM.
(EPS)
